# Management System, Resource Constraints, and Composite Disease Burden in Dromedary Camels in El Oued, Algeria: An Observational Study with Exploratory Dystocia Analyses

**DOI:** 10.3390/ani16203303

**Published:** 2026-10-20

**Authors:** Mohamed Amine Fares, Mohamed Tharwat, Randa Mlik, Carlos Iglesias Pastrana, Ahmed Mennai, Aicha Mouane, Sulaiman F. Aljasir

**Affiliations:** 1 Higher School of Saharan Agriculture, El Oued PB90, El Oued 39000, Algeria; fares.mohamedamin@esas-eloued.dz (M.A.F.); mlik.randa@esas-eloued.dz (R.M.); mennai.ahmed@esas-eloued.dz (A.M.); 2 Saharan Area Laboratory for Agricultural Modernization and Advancement (SALAMA LAB), Higher School of Saharan Agriculture, El Oued PB90, El Oued 39000, Algeria; 3 Department of Animal Medicine, Faculty of Veterinary Medicine, Zagazig University, Zagazig 44519, Egypt; 4 The Andalusian Institute for Agricultural, Fishing, Food and Ecological Production Research and Training (IFAPA), Centro Alameda del Obispo, 14004 Cordoba, Spain; 5 Laboratory of Biodiversity and Application Biotechnology in Agriculture, Department of Biology, Faculty of Nature and Life Sciences, University of El Oued, El Oued 39000, Algeria; aicha-mouane@univ-eloued.dz; 6 Department of Veterinary Preventive Medicine, College of Veterinary Medicine, Qassim University, P.O. Box 6622, Buraydah 51452, Saudi Arabia

**Keywords:** *Camelus dromedarius*, animal welfare, sedentarization, halophytes, disease burden, dystocia, veterinary epidemiology, Sahara, Algeria

## Abstract

**Background**: Dromedary camel husbandry in Algeria is shifting toward sedentary confinement, yet the consequences for herd health and reproductive outcomes remain poorly documented. This study examined the associations of housing system, environmental constraints, and disease burden with dystocia in El Oued, Algeria. **Methods**: A 12-month observational study of 55 farms (45 extensive, 10 semi-intensive) and 70 focal adult females integrated surveys, clinical assessments, and retrospective records. Herd Total Disease Burden (TDB; 0–24 scale) was modeled using negative-binomial regression (n = 55). Exponentiated coefficients were interpreted as ratios of expected TDB scores. **Results**: Feed, water, and health constraints were strongly correlated (Spearman’s ρ = 0.89–0.96). Semi-intensive management yielded higher expected TDB scores than extensive management (ratio = 1.71, 95% CI: 1.28–2.29, *p* < 0.001), as did feed (ratio = 1.15, *p* = 0.015) and water deficits (ratio = 1.10, *p* = 0.048). Confined herds reported greater forage-species richness and higher female body weights; however, current individual clinical morbidity positively correlated with historical dystocia rates. An exploratory Random Forest ranked herd size and TDB as top farm-level dystocia predictors, though its out-of-bag error (25.5%) exceeded the 16.4% error of the majority-class baseline. **Conclusions**: Semi-intensive management and resource constraints are associated with higher composite disease-burden scores. Forage-species richness and increased body weight alone should not be interpreted as indicators of better health. The exploratory classifier underscores the need for larger studies to validate predictive reproductive models.

## Simple Summary

Camel herders in the Souf region of El Oued, Algeria, are increasingly keeping their one-humped camels in pens instead of moving them across the desert in search of grazing. This change makes feeding and watering easier to manage, but it is not clear whether it helps or harms the animals’ health. To find out, we followed 56 camel farms for a year, 45 where camels roam freely, 10 partly confined, and 1 fully confined, and examined 80 breeding females, alongside farm records of illness and difficult births. Camels kept partly indoors had higher observed disease-burden scores than those allowed to roam freely, and herds that did not receive enough feed or water were also sicker. Offering a wider variety of plants did not compensate for the problems linked to confinement. Females that had previously experienced a difficult birth were more likely to be ill, and heavier confined females were not necessarily healthier. Our findings indicate that confining camels without sufficient feed and water was associated with higher disease-burden scores; its relationship with reproductive outcomes requires further investigation. Herders and veterinarians can use these results to improve how confined camels are fed, watered, and monitored, supporting both animal welfare and the livelihoods that depend on these animals.

## 1. Introduction

The dromedary camel represents the physiological and socio-economic keystone of hyper-arid pastoral ecosystems across North Africa and the Middle East [1,2]. The species’ evolutionary adaptations, efficient urea recycling, extremely low water turnover, tolerance of substantial hemoconcentration, and the highly selective browsing of halophytic and thorny vegetation were optimized within traditional pastoral production systems governed by extensive spatial mobility [3,4]. In the Northern Algerian Sahara, extensive dromedary herds exploit the spatial and temporal heterogeneity of pastures dominated by perennial halophytes and psammophytes of the Chenopodiaceae, Poaceae, and Fabaceae families [5]. This botanical complex supplies the essential electrolytes, macro-minerals, and trace elements required for the unique metabolic homeostasis of the species [6,7].

Over the past two decades, demographic expansion, rangeland fragmentation, recurrent severe droughts, and national agricultural development policies promoting groundwater-irrigated agriculture have accelerated the sedentarization of Saharan pastoralists [8,9]. In southeastern Algeria, particularly in the Souf region, traditional transhumant and nomadic systems are rapidly transitioning along a structural continuum toward semi-intensive enclosures and peri-urban sedentary units [10,11]. Recent field evidence from the El Oued camel sector likewise indicates that climate extremes, forage scarcity, shelter quality, water management, and veterinary-care limitations interact within the local husbandry system, reinforcing the importance of treating environmental and management constraints as interconnected rather than isolated exposures [12].

Although confinement and production intensification facilitate market integration, mechanized milking, and reproductive management, they also modify the housing, feeding, behavioral, health, and reproductive conditions of dromedaries, making welfare an important consideration in increasingly intensive production systems [13,14]. Behavioral activity and time allocation are influenced by management conditions in dromedaries, and recent work in intensively managed dairy camels has shown that restricted housing is associated with characteristic daily activity budgets, including relatively little walking and substantial periods devoted to feeding, rumination, standing, and lying [15]. Concurrently, confinement may alter disease transmission ecology. Enclosed paddocks and elevated stocking densities may contribute to the transmission and persistence of contagious ectoparasites, including *Sarcoptes scabiei* var. *cameli* and *Hyalomma* ticks, particularly where animals have prolonged contact with shared environments [16,17]. Furthermore, substituting diverse halophytic rangeland browse with monotonous, energy-dense cultivated rations can induce subclinical mineral imbalances (particularly copper, zinc, and selenium) that severely compromise immune competence [18,19,20]. Importantly, camel immune tissues may themselves reflect these husbandry contrasts: comparative work in El Oued demonstrated significant differences in the morphology and cellular composition of mesenteric lymph nodes between free-roaming and restricted-roaming rearing systems, supporting the biological plausibility that management conditions can modify host immune status [21].

The reproductive repercussions of these profound management shifts warrant critical examination. Dromedary reproduction is intrinsically conservative, characterized by late puberty, seasonal induced ovulation, a 12-to-13-month gestation, and extended intercalving intervals [22]. Dystocia represents a severe, life-threatening obstetric emergency in the dromedary, risking maternal pelvic trauma, uterine rupture, secondary prolapse, and fetal mortality [23,24]. Established individual-level risk factors include primiparity, fetal maldisposition, pelvic disproportion, and maternal exhaustion [25,26]. However, the systemic, upstream contribution of farm-level health indicators—specifically the combined influence of housing type, nutritional constraints, and cumulative disease burden—remains insufficiently quantified in transitioning Saharan systems.

This study investigated camel production systems across the extensive–intensive spectrum in the El Oued region of southeastern Algeria. Our primary objectives were: (1) to characterize farm typologies across demographic, management, and health domains using multivariate ordination; (2) to quantify the farm-level associations between housing systems, environmental constraints, and herd disease burden using appropriate count regression modeling; (3) to evaluate the floristic composition of native rangeland browse utilized by mobile pastoralists; and (4) to examine exploratory predictors of farm-level dystocia occurrence. We hypothesized that management intensification is associated with elevated herd disease burdens, and that higher cumulative morbidity and increasing herd scales are positively associated with the occurrence of obstetric dystocia.

## 2. Materials and Methods

### 2.1. Study Area

The study was conducted across the pastoral and agricultural zones of the wilaya of El Oued (33°22′ N, 6°52′ E; 60–80 m above sea level), located within the Souf basin of the Northern Algerian Sahara. The study area is located in the hyper-arid Souf basin of the Northern Algerian Sahara. Regional climatic conditions were characterized using historical climatological data for the 1991–2020 reference period, obtained from the World Bank Group’s Climate Change Knowledge Portal (World Bank Group, Washington, DC, USA; incorporating CRU TS observational climatologies [Climatic Research Unit, Norwich, UK] and ERA5 reanalysis [European Centre for Medium-Range Weather Forecasts, Reading, UK]). Based on these historical records, mean annual precipitation rarely exceeds 80 mm, and the region experiences extreme thermal amplitudes, with summer maxima routinely exceeding 45 °C and winter minima approaching 0 °C [27]. Geomorphological features are defined by the active sand dunes of the Grand Erg Oriental, endorheic saline depressions (chotts), and unique interdune agricultural hollows (ghouts) where traditional date-palm cultivation occurs [28]. The region supports one of the most significant dromedary populations in Algeria, predominantly of the Sahraoui pastoral ecotype [10].

### 2.2. Study Design and Sampling Framework

A 12-month observational study comprising a farm-level baseline survey and repeated animal-level clinical assessments was conducted between October 2024 and September 2025. To ensure an analytically appropriate structure, the study utilized a strictly separated two-tiered hierarchical sampling design.

Tier 1—Farm-Level Baseline (n = 55): Camel herds were enrolled across the agricultural districts (daïras) of El Oued using stratified purposive sampling to capture the full spectrum of local husbandry systems. Inclusion criteria mandated: (i) a minimum herd size of 10 adult camels, (ii) continuous operation under the designated management system for at least the three preceding years, and (iii) informed owner consent. Farms were classified into three production systems (Table 1):

- Extensive (Nomadic/Transhumant; n = 45): Herds grazed open desert rangelands year-round without permanent perimeter fencing or housing infrastructure. Supplemental feeding was limited and was provided primarily during periods of pronounced seasonal forage scarcity.
- Semi-Intensive (n = 10): Herds were enclosed overnight or seasonally within fenced paddocks, permitted daily or seasonal grazing access on peri-urban rangelands, and received regular nutritional supplementation (e.g., cultivated green alfalfa, barley grain, dry dates, agricultural by-products).
- A single intensive commercial holding (permanent pen confinement, 100% cultivated feed) was initially observed but was strictly excluded from all statistical analyses, summary tables, and data visualizations because a sample size of one precludes variance estimation and introduces analytical bias. Thus, the final analytical sample comprised 55 farms.

Tier 2—Animal-Level Longitudinal Monitoring (n = 80 females): Within each enrolled herd, focal adult breeding females (≥5 years of age, having completed at least one prior pregnancy) were randomly selected for clinical monitoring. One focal female was enrolled per extensive herd (with 5 larger extensive herds contributing 2 females, total n = 50) and two focal females per semi-intensive herd (n = 20). Each focal female was clinically evaluated across three prospective seasonal visits (Visit 1: autumn/winter; Visit 2: spring calving peak; Visit 3: late summer dry season).

For the 70 focal females, age, parity, and available reproductive history were recorded from detailed structured owner interviews cross-referenced with available herd veterinary records. The number of previous pregnancies and calvings, the number of recorded dystocia events, and the completeness of the reproductive records were summarized before the DBI–dystocia analysis. Missing information was handled by strict exclusion; animals without an ascertainable denominator for the historical individual dystocia rate were not assigned an assumed value and would have been excluded, though complete baseline reproductive histories were successfully obtained for all 80 enrolled females. The detailed reproductive denominators are provided in Supplementary Table S2.

Field interviews and clinical evaluations were conducted by the same two veterinary investigators throughout the study period to ensure diagnostic consistency and minimize interobserver variation.

### 2.3. Clinical Case Definitions and Variable Operationalization

Farm-level constraint scores for feed availability, water availability, herd health, and reproductive management were recorded using a four-category ordinal scale (0–3). Ratings were based on structured interviews with herd owners and corroborated, where possible, by direct farm inspection. Increasing scores represented increasing severity and/or frequency of the reported constraint. The specific indicators used to assign each score differed by domain and are described in Table 2. These scores represented semi-quantitative field assessments of the owner’s operational challenges rather than direct measurements of feed intake, water consumption, or clinical disease incidence. Importantly, the owner-perceived health constraint score was recorded independently of the clinically assessed Total Disease Burden (TDB), and the reproductive constraint score evaluated conception and gestation issues explicitly excluding dystocia to prevent analytical circularity.

Herd Total Disease Burden (TDB) was calculated as the sum of the ordinal scores assigned to eight clinically defined syndromes for each farm. Each syndrome received a single score from 0 to 3: 0 indicated absence; 1 indicated sporadic occurrence or mild manifestations; 2 indicated moderate or endemic occurrence; and 3 indicated a severe or generalized outbreak. The total score was calculated as TDB = Σs_j_, where s_j_ is the score assigned to syndrome j and j ranges from 1 to 8. The theoretical range was therefore 0–24. Prevalence and clinical severity were considered within the syndrome-level classification rather than being treated as separate numerical components of the total score.

All eight syndromes were given equal weight to obtain a transparent summary of the farm-level clinical burden. The index was not intended to imply that the syndromes have equal biological severity or welfare impact. It should be interpreted as an exploratory composite measure of the recorded syndromic burden rather than as a validated measure of total disease incidence or overall animal welfare. The consequences of equal weighting should be considered when interpreting associations with management variables. The specific criteria used to assign the 0–3 scores for each syndrome are detailed in Supplementary Table S1.

The clinical entities were diagnosed according to standard camelid veterinary protocols [20,29]:

- Sarcoptic Mange: Scored based on the presence of intense pruritus, extensive alopecia, and severe hyperkeratosis/crusting characteristic of *Sarcoptes scabiei* var. *cameli*.
- Tick Infestation: Scored by the macroscopic density and clustering of *Hyalomma* spp. on the perianal, axillary, sternal, and inguinal regions.
- Diarrheal Enteritis Syndrome: Profuse watery or mucoid feces, severe perineal soiling, and signs of clinical dehydration.
- Respiratory Disease Complex: Serous to mucopurulent nasal discharge, spontaneous coughing, tachypnea/dyspnea, and abnormal pulmonary auscultation.
- Contagious Skin Necrosis/Abscesses: Subcutaneous nodular abscessation (often associated with Corynebacterium or Streptococcus complexes) and peripheral lymphadenitis.
- Musculoskeletal/Lameness: Observable locomotor impairment, joint effusion, severe foot-pad ulceration, or chronic gait asymmetry preventing normal herd movement.
- Ocular Afflictions: Acute or chronic keratoconjunctivitis, corneal opacity, or purulent lacrimation.
- Wasting Syndrome/Cachexia Syndrome: Progressive loss of body condition, temporal muscle atrophy, pale mucous membranes, and intermittent pyrexia. This category was recorded as a clinical syndromic phenotype rather than a confirmed etiological diagnosis. Because pathogen-specific parasitological or molecular confirmation was not performed, affected animals were not classified as confirmed cases of trypanosomosis.
- At the animal level, for each focal female, the Individual Disease Burden Index (DBI) was calculated by normalizing the sum of the eight syndrome-specific clinical severity scores to a 0–100 scale: DBI = 100 × (Σs_j_/24), where s_j_ is the score for syndrome j (0–3) and the maximum possible sum is 8 × 3 = 24. Higher DBI values indicate a greater composite burden of the recorded clinical syndromes. Estimated Live Body Weight (kg) was calculated using validated dromedary barymetric equations utilizing thoracic girth, abdominal girth, and shoulder height measured using a standard flexible measuring tape [30]. Because body size is inherently multidimensional in dromedaries, interpretation of estimated live weight was complemented by consideration of documented size-related morphological variation in the species [31]. For this study, dystocia was operationally defined on the basis of the reported or documented need for obstetric intervention. A parturition was classified as dystocia when the owner report or available herd records indicated that physical traction, fetotomy, or caesarean section was required to complete delivery. Farm-level dystocia occurrence was defined as a binary indicator, coded positive when at least one qualifying dystocia event requiring physical traction, fetotomy, or caesarean section was reported or documented within the preceding 12 months. This outcome describes whether a farm experienced at least one recorded event; it does not estimate the proportion of calvings complicated by dystocia. The number of births per farm was not incorporated into this binary definition. Because the outcome was reconstructed retrospectively from owner reports and available records, classification did not rely on a uniform duration-of-labor threshold. Prolonged labor or an abnormal fetal presentation, position, or posture alone was not sufficient for classification under the study definition unless the event met the specified intervention criterion. This operational definition was informed by published clinical reports of dystocia in dromedary camels [25,26].
- Individual dystocia rate (%) was calculated as the percentage of the focal female’s historical lifetime calvings requiring one of the specified obstetric interventions, based on owner recall and available herd records [25,26].

### 2.4. Botanical Inventory and Pastoral Valuation

Dietary plant richness was assessed using the field records for the enrolled farms. In extensive farms, herders identified plant taxa actively browsed on natural rangelands using local vernacular names, and specimens were identified by matching morphological characteristics with regional Saharan taxonomic keys and established forage inventories [5,28]. In semi-intensive and intensive farms, the number of distinct plant taxa and feed ingredients recorded as consumed was determined from structured interviews with the herd owner detailing the daily provided rations (e.g., cultivated green alfalfa, barley grain, dry dates, and agricultural by-products) alongside any seasonal peri-urban grazing access. Plant richness was defined as the total number of distinct taxa or feed components recorded for each farm. This measure represented taxonomic richness only and did not quantify dietary biomass, dry-matter intake, feeding frequency, nutrient balance, or the nutritional adequacy of the ration. The botanical identification procedures and scope of assessment differed according to the production system and are described accordingly.

### 2.5. Statistical Analyses

All data processing and statistical modeling were performed in R (version 4.3.3; R Foundation for Statistical Computing, Vienna, Austria; https://www.R-project.org/). Statistical significance was pre-specified at a two-tailed α = 0.05.

Conceptual Framework: Before statistical modeling, a conceptual directed acyclic graph (DAG) was used to organize the hypothesized relationships among housing system, environmental constraints (feed and water deficits), herd size and crowding, cumulative disease burden, and dystocia occurrence. The framework distinguished the primary management exposure from potential intermediate variables and demographic factors that could be associated with the outcomes. The DAG was used to structure the observational analyses and communicate the hypothesized relationships among variables; it was not treated as evidence of causality.

The conceptual framework was used to organize the hypothesized relationships among management system, environmental constraints, herd size, disease burden, and dystocia. These relationships were not assumed to be causal effects established by the data. In the primary farm-level regression, management system, feed-deficit severity, water-deficit severity, and standardized herd size were entered simultaneously. Therefore, the management-system coefficient represents its conditional association with TDB given the other included predictors, rather than the total association of management system with TDB. Because feed and water constraints may lie on pathways linking management practices to disease burden, adjustment for these variables may change the interpretation of the management-system coefficient.

Correlation and Bivariate Structures: Spearman’s rank correlation coefficients (*ρ*) were calculated for the farm-level variables included in the correlation analysis (n = 55): herd size (continuous), weaning age (continuous), dietary plant diversity (count), Total Disease Burden (TDB score, 0–24), feed-deficit severity (0–3), water-deficit severity (0–3), owner-perceived health constraint (0–3), reproductive constraint excluding dystocia (0–3), and binary farm-level dystocia occurrence. The correlation matrix was used to describe pairwise monotonic associations among the recorded management, resource-constraint, health, and reproductive variables. Complete data were available across all 55 holdings for these parameters, ensuring all coefficients were calculated from the identical denominator. Pairwise associations and continuous distributions across systems were evaluated using generalized pairs plots.

Multivariate Farm Typologies: Multiple Factor Analysis (MFA) was performed using the FactoMineR package (version 2.9; R Foundation for Statistical Computing) on 55 farms (45 extensive and 10 semi-intensive), excluding the single intensive holding. Variables were organized into five thematic groups: System, Demography, Management, Constraints, and Health/Flora. The System group comprised the categorical housing system; Demography comprised total herd size; Management comprised weaning age; Constraints comprised feed-deficit severity and water-deficit severity; and Health/Flora comprised the Total Disease Burden (TDB) score and dietary plant diversity. Variable types were identified as quantitative or categorical as appropriate. Quantitative variables were centered and scaled to unit variance (type “s”), and the categorical variable was coded as a nominal active variable (type “n”). Group weighting followed the default FactoMineR procedure, where variables are weighted by the inverse of the first principal component eigenvalue of their respective group. All seven aforementioned variables were designated as active, and no supplementary variables were used. Missing values were absent in this subset, as missing field indicators were coded as zero during data curation. The first two dimensions were retained for graphical interpretation, with their percentages of total inertia reported in the Results.

Primary Inferential Modeling for Disease Burden: The primary outcome was farm-level Total Disease Burden (TDB), an additive composite score ranging from 0 to 24, calculated by summing the syndrome-specific ratings for eight clinically defined syndromes, each scored from 0 to 3. TDB represents a composite measure of syndrome occurrence and severity rather than a count of individual disease cases or a disease-incidence rate per animal. Because the TDB composite score exhibited substantial overdispersion (variance > mean) and a right-skewed distribution with zero-inflation, a negative-binomial generalized linear model was selected over a standard Poisson or Gaussian model to prevent underestimation of standard errors. Herd size was entered as an estimated covariate to assess its association with the composite score, rather than as an exposure offset. Because the outcome was not defined as a count of individual disease events per population at risk, no log(herd size) offset was included. Exponentiated coefficients were interpreted as ratios of expected TDB scores, not incidence rate ratios.

ln(𝔼[TDB*_i_*]) β_0_ + β_1_(System*_i_*) + β_2_(FeedDeficit*_i_*) β_3_(WaterDeficit*_i_*) β_4_(HerdSize(std)*_i_*) (1)

Exponentiated regression coefficients (exp(β)) and 95% Wald confidence intervals were extracted. The exponentiated coefficients were interpreted as ratios of expected TDB scores rather than incidence rate ratios.

Collinearity assessment and sensitivity analyses: Pairwise associations among the predictors considered for the primary farm-level model were examined using Spearman’s rank correlation coefficients. Collinearity among the fitted predictors was assessed using generalized variance inflation factors (GVIFs). To address potential instability arising from highly correlated environmental constraints, sensitivity analyses evaluated the stability of the estimated associations when feed deficit and water deficit were considered in separate model specifications. Estimates, confidence intervals, and model diagnostics were explicitly compared across these specifications to confirm the robustness of the primary full model.

Animal-Level Associations: Although each focal female was clinically assessed at three prospective seasonal visits, the analytical objective was to evaluate cumulative chronic morbidity rather than fine-scale temporal variation. Therefore, the clinical observations from the three visits were aggregated to generate a single summary Individual Disease Burden Index for each female, representing her overall clinical status across the study period.

Association with Historical Dystocia: The association between the focal female’s clinical disease burden and her historical individual dystocia rate was evaluated separately using Spearman’s rank correlation, with the female-level summary of clinical DBI used for this analysis. Because historical dystocia rate represents cumulative reproductive history rather than a repeated prospective outcome, it was not treated as a repeated-measures variable. Because focal females were sampled from multiple farms, the association between individual DBI and historical dystocia rate was reassessed using a farm-clustered sensitivity analysis. Farms, rather than individual females, were treated as the resampling units (1000 bootstrap iterations) to account for the non-independence of females sampled from the same holding. The cluster-adjusted uncertainty estimates were compared with those from the original individual-level Spearman correlation.

Exploratory Dystocia Analyses: Because farm-level dystocia occurred on a limited subset of holdings (10 of 55 farms, 17.9%), parametric multivariable logistic regression risked severe small-sample bias. We applied a non-parametric Random Forest classifier (package (version 4.7-1.1; R Foundation for Statistical Computing; *n*_tree_ = 500, *m*_try_ = 2), *n*_tree_ = 500, *m*_try_″ = 2) across all 55 farms to explore variable importance rankings for dystocia occurrence. Because 46 of the 55 farms recorded no dystocia, a naive majority-class classifier predicting “No” for all instances would achieve a baseline error rate of 17.9%. Model stability was evaluated via internal out-of-bag error and compared against this naive baseline to determine the robustness of the classification. This procedure was utilized strictly for exploratory pattern prioritization rather than causal inference.

The analytical sample size varied according to the analysis. The Multiple Factor Analysis and primary negative-binomial model of farm-level Total Disease Burden included 55 farms (45 extensive and 10 semi-intensive), excluding the single intensive holding because that management category lacked farm-level replication. Farm-level bivariate correlations and the exploratory Random Forest analysis of dystocia occurrence used all 55 holdings. The animal-level analyses included all 70 focal females, including the ten females sampled from the intensive holding. Accordingly, the multivariate and primary disease-burden models describe associations within extensive and semi-intensive systems, whereas findings from analyses including the intensive holding are potentially influenced by that single farm and do not constitute a replicated intensive-system comparison.

## 3. Results

### 3.1. Conceptual Analytical Framework

**Figure 1:**
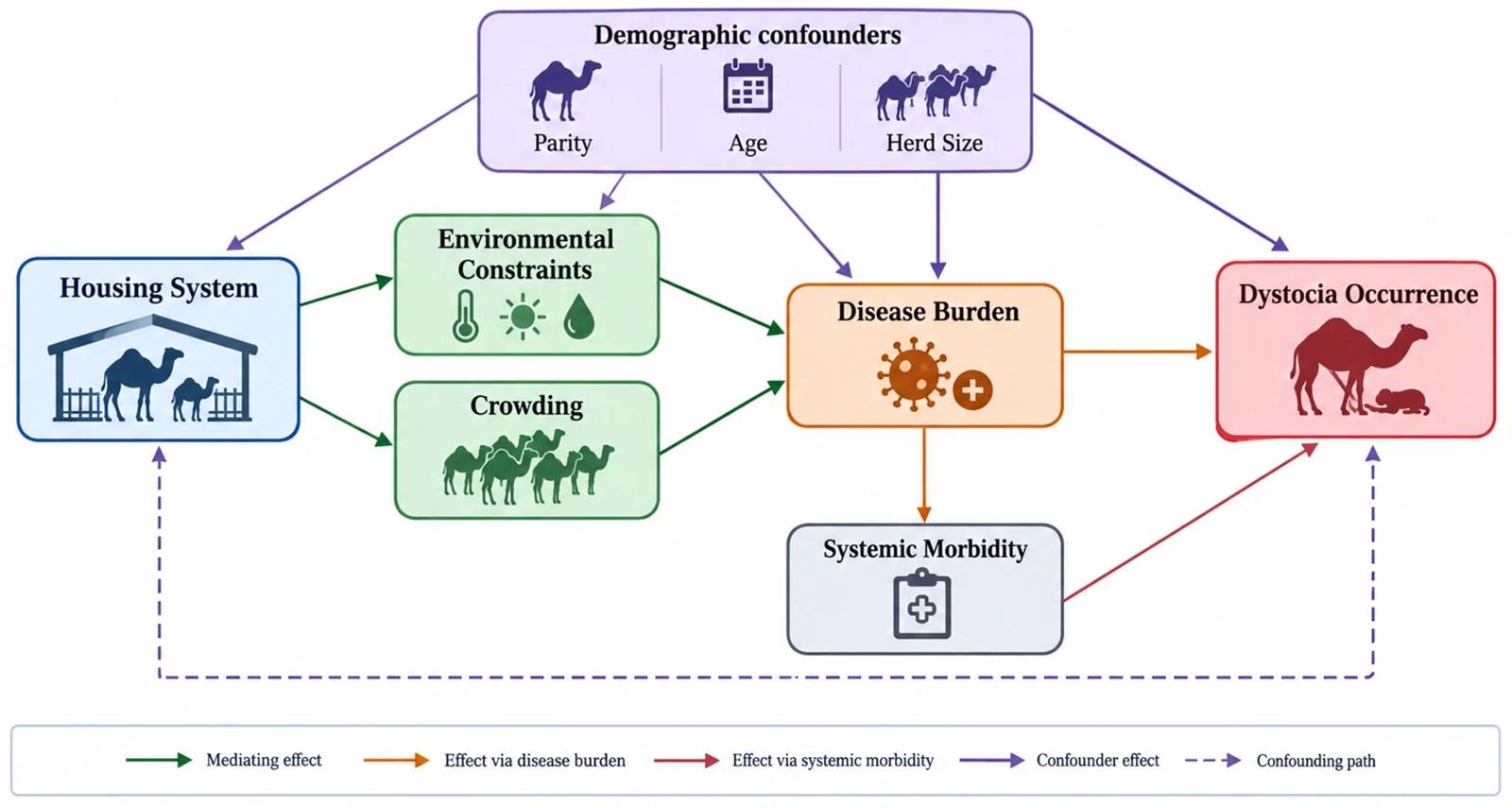
Conceptual analytical framework. Directed Acyclic Graph used to organize the observational analyses. Housing System acts as the primary exposure, associated with downstream Environmental Constraints and Crowding. These mediators converge on Disease Burden. Dystocia Occurrence is evaluated as the final clinical outcome, influenced by systemic morbidity alongside demographic confounders such as Parity, Age, and overall Herd Size. This framework organizes the statistical sequence without claiming strict causal proof of intervention effects.

Figure 1 presents the Conceptual Analytical Framework utilized to organize the observational analyses. This diagram distinguishes the primary structural exposure from intermediate environmental constraints and downstream morbidity indices, acknowledging the presence of demographic confounders.

### 3.2. Bivariate Constraint Correlations

Spearman correlation analysis demonstrated an intense, tightly interlocking block of environmental and owner-perceived constraint scores across the 55 holdings. Perceived feed deficits correlated exceptionally strongly with subjective health constraints (ρ = 0.96, *p* < 0.001) and watering constraints (ρ = 0.93, *p* < 0.001). The clinically observed herd TDB score showed a distinct positive correlation with the subjective owner-perceived health constraint, which suggests concordance between clinical and caretaker-based assessments.

**Figure 2:**
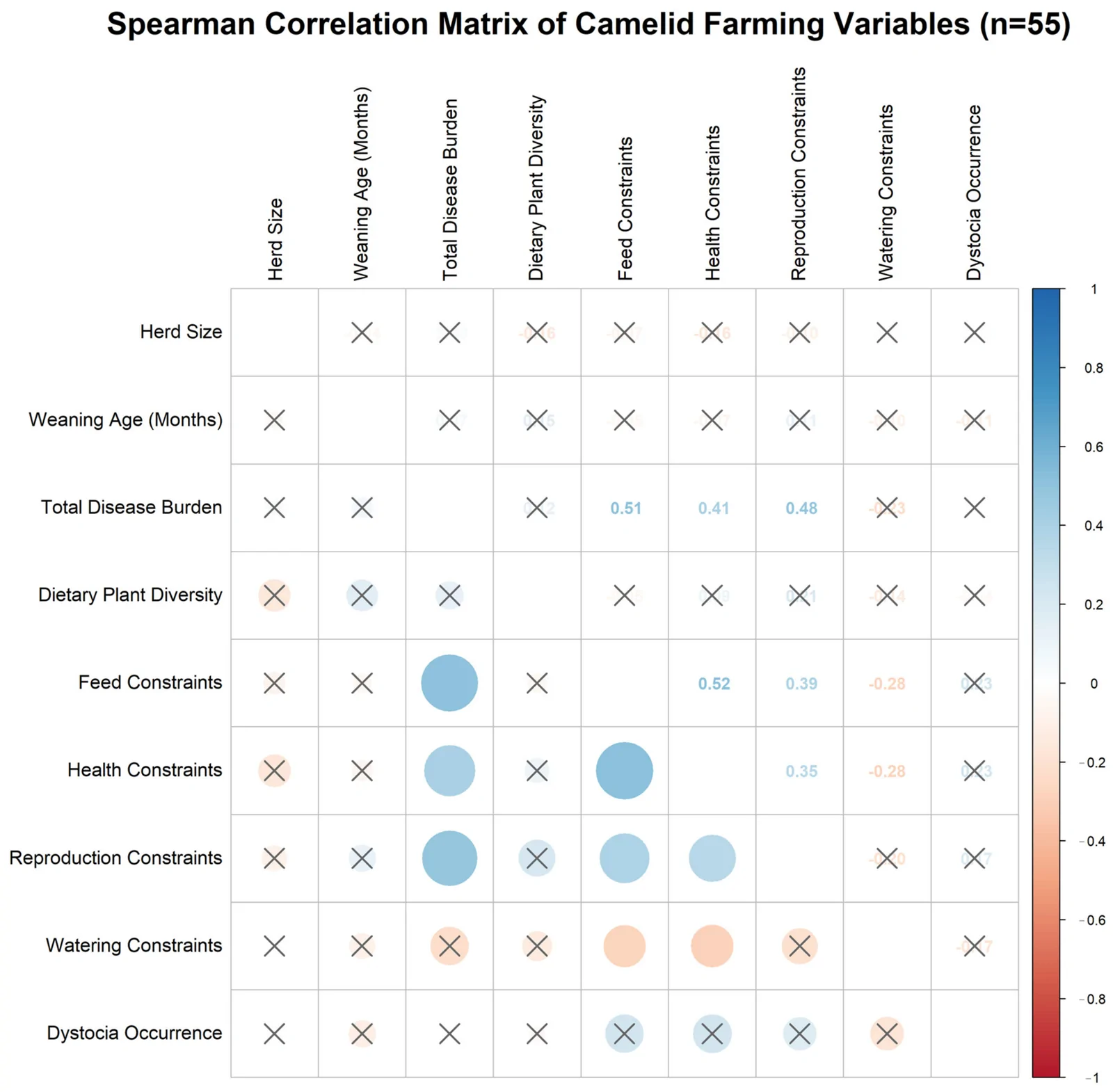
Spearman rank correlation matrix of farm-level parameters. The correlogram highlights the near-collinear relationship between feed, water, and health constraints, alongside the positive correlations linking Total Disease Burden and Dystocia Occurrence. Non-significant correlations (*p* ≥ 0.05) are marked with an ‘X’.

As illustrated in Figure 2, farm-level binary dystocia occurrence did not demonstrate statistically significant bivariate monotonic correlations with TDB (Spearman’s *ρ* = 0.041, *p* = 0.763), water-constraint severity (*ρ* = −0.216, *p* = 0.110), or feed-constraint severity (*ρ* = 0.254, *p* = 0.059). Because binary farm-level occurrence is heavily confounded by herd size and lacks a precise calving denominator, these simple bivariate associations do not capture the full clinical picture. Consequently, the relationship between dystocia and disease burden was further evaluated using both multivariate exploratory classification (Section 3.7) and mathematically robust individual-level clinical data (Section 3.6).

### 3.3. Farm Typology and Multivariate Structure

**Figure 3:**
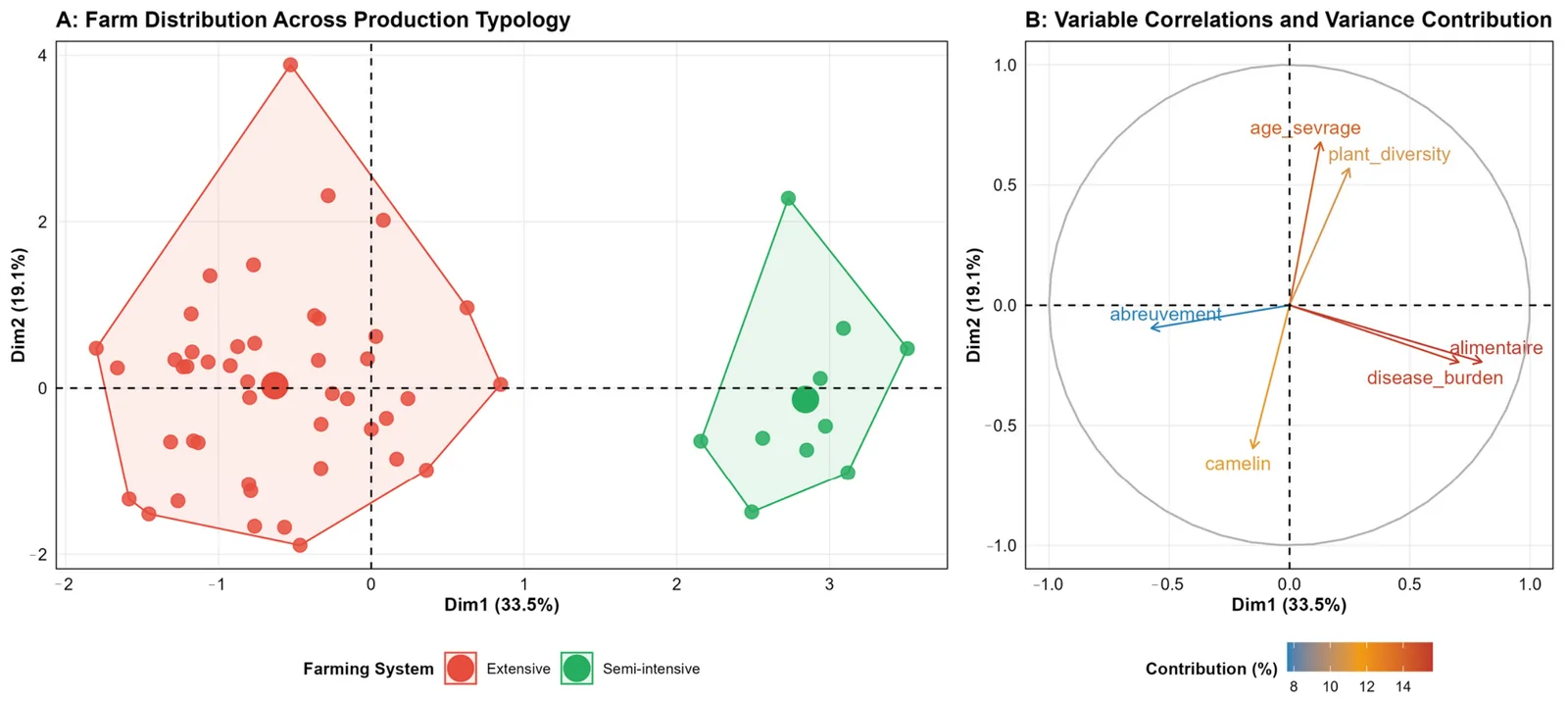
Multiple Factor Analysis (MFA) of 55 camel farms. (**A**) Farm scores on Dimensions 1 and 2, accounting for 33.5% and 19.1% of total inertia, respectively. Points represent plotted farms, colored by management system (extensive, n = 45; semi-intensive, n = 10); polygons outline the distribution of farm scores within each group. (**B**) Variable correlation and contribution plot for the first two dimensions. Arrows indicate the directions of variable vectors, and arrow colors represent variable contributions to the axes, as indicated by the color scale.

In the farm-level MFA (n = 55 holdings; Figure 3), Dimension 1 accounted for 33.5% of total inertia and Dimension 2 accounted for 19.1%. Dimension 1 served as an explicit constraint and morbidity axis, driven strongly by herd TDB, feed constraints, and watering constraints (all loading deeply on the positive axis).

The MFA plot showed a visual separation between the extensive and semi-intensive farms along Dimension 1, with extensive farms predominantly occupying negative coordinates and semi-intensive farms occupying positive coordinates. This pattern describes the configuration of the sampled farms in the reduced-dimensional space and does not, by itself, constitute a formal statistical test of differences between management systems. Furthermore, the plotted distribution was characterized by differences in the recorded management, constraint, and health variables across the two sampled groups. These patterns are descriptive and should be interpreted in conjunction with the univariate and regression analyses.

**Figure 4:**
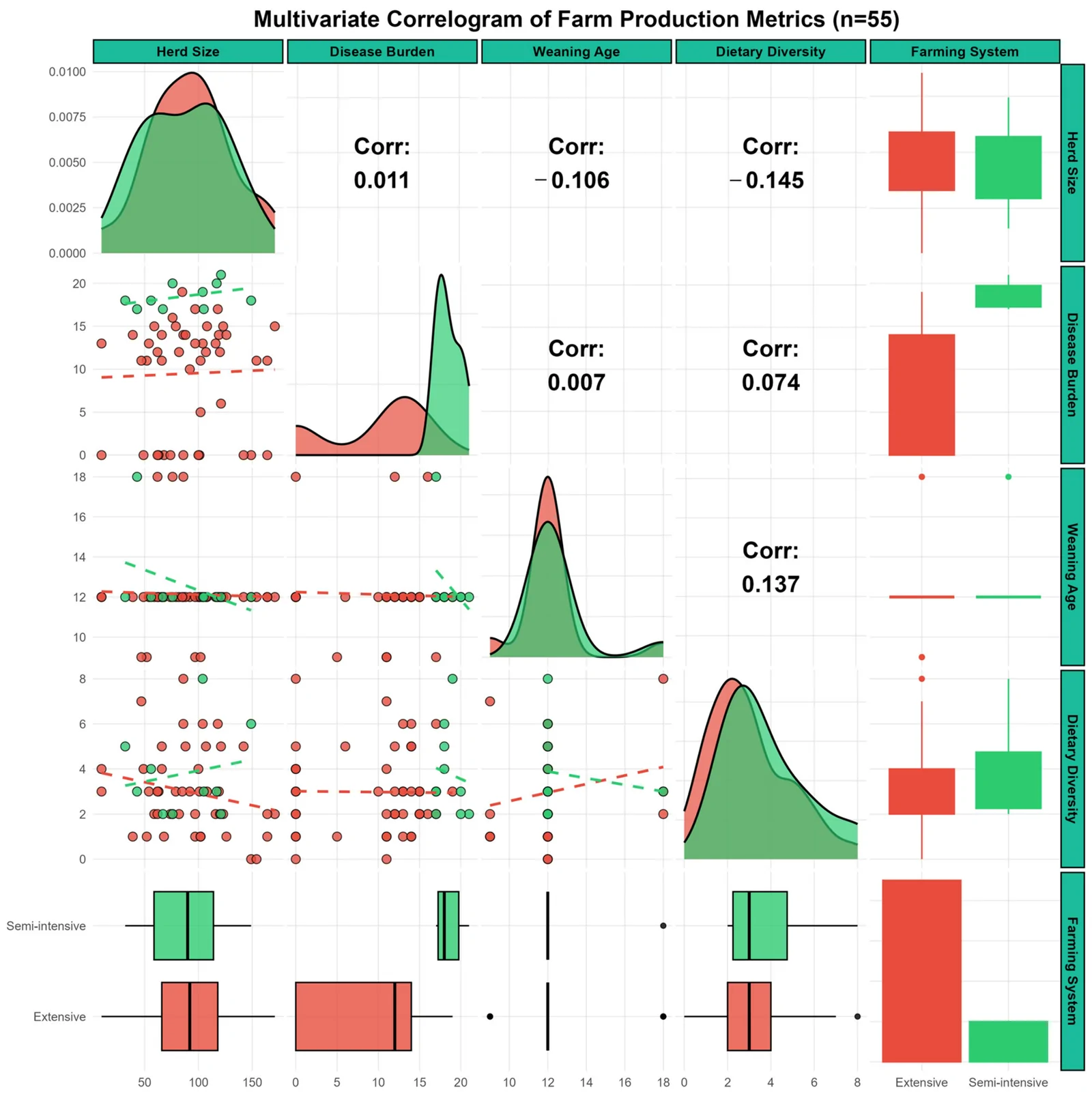
Multivariate distributions and pairwise relationships of farm production metrics (*n* = 55). Diagonal density plots, scatter plots with fitted trend lines, and box plots are color-coded by management system: red represents extensive farms (*n* = 45) and green represents semi-intensive farms (*n* = 10).

The generalized pairs plot further illuminated these structural divergences, confirming a severe right shift in disease burden for semi-intensive systems compared to the bimodal, heavily zero-inflated distribution of extensive herds (Figure 4).

### 3.4. Botanical Rangeland Composition

Sixteen native pastoral species were recorded on the 45 extensive farms. Table 3 presents the seven most frequently cited taxa utilized by the pastoralists. The botanical foundation of nomadic herds was dominated by two halophytic Chenopodiaceae—*Anabasis articulata* (Baguel; 73.3% of holdings) and *Traganum nudatum* (Demranne; 55.6%)—alongside the fibrous perennial tussock grass *Stipa tenacissima* (Helfa; 53.3%). A secondary tier included *Limoniastrum guyonianum* (44.4%) and *Stipagrostis* complexes (40.0%).

Interestingly, the median extensive herd reported relying on only three core species. In contrast, semi-intensive farms reported a higher raw count of consumed species (median 5). This difference may reflect access to heterogeneous peri-urban vegetation and supplementary feeds; however, species counts alone do not quantify dietary biomass, intake, seasonal complementarity, or nutrient balance.

### 3.5. Primary Farm-Level Predictors

Among the 55 farms included in the primary regression analysis, the observed TDB score had a mean of 11.1 (SD 6.7), a median of 13 (IQR 8–15.5), and a range of 0–21. A total of 12 farms (21.8%) had a TDB score of zero.

**Figure 5:**
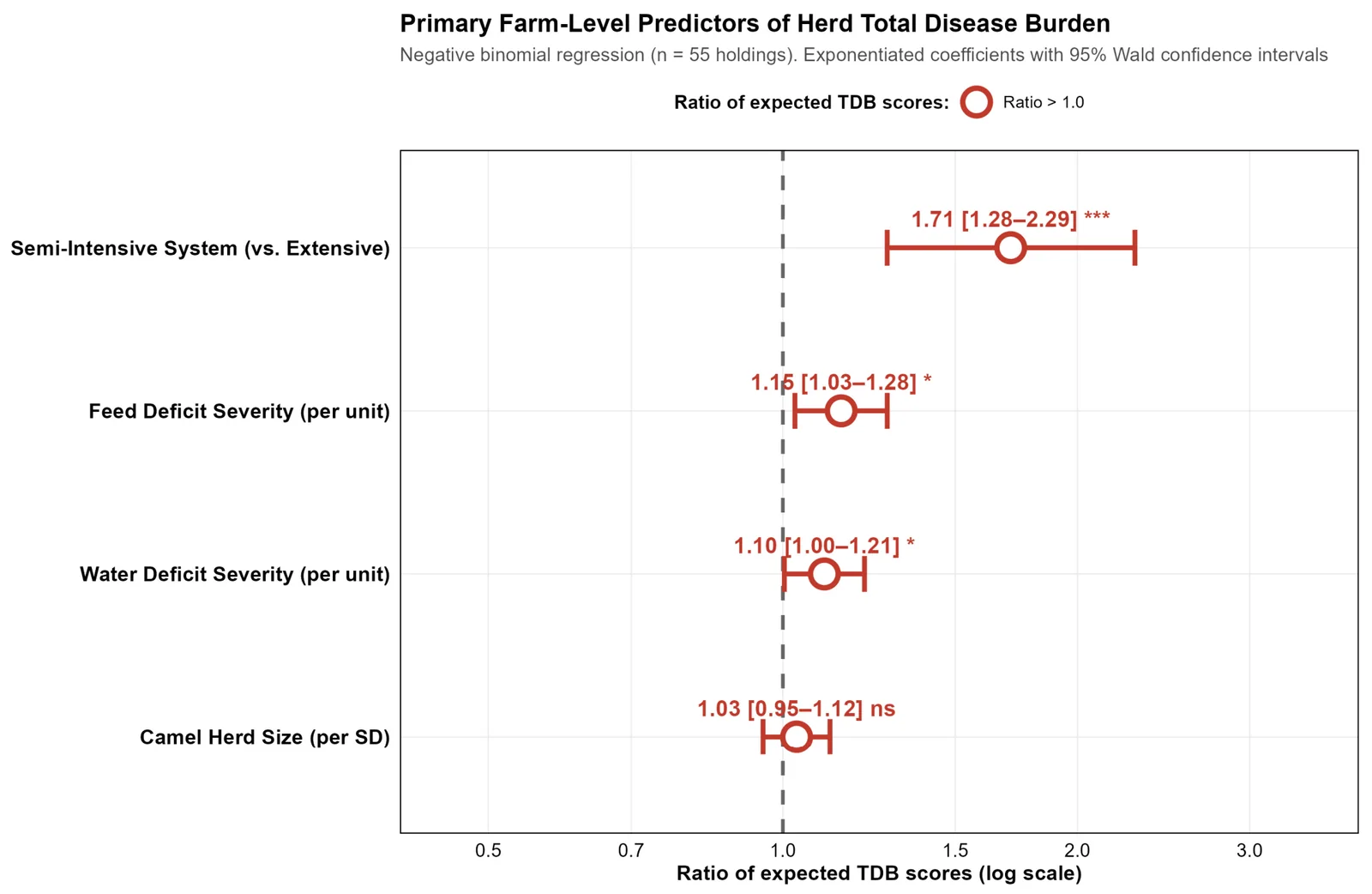
Negative-binomial regression of farm-level Total Disease Burden (TDB; n = 55 farms). Points represent exponentiated regression coefficients, and horizontal bars represent 95% Wald confidence intervals for the associations of management system, feed-deficit severity, water-deficit severity, and standardized herd size with the expected TDB score. The vertical dashed line indicates a ratio of 1. Extensive management is the reference category for the management-system comparison. Statistical significance levels: * *p* < 0.05, *** *p* < 0.001, ns = non-significant (*p* ≥ 0.05).

The primary negative binomial regression model of herd TDB, fitted at the true observational unit of the farm (n = 55), identified housing and nutritional constraints as significant predictors of clinical morbidity (Table 4; Figure 5).

Semi-intensive management was associated with an expected TDB score 1.71 times that of extensive management (ratio of expected TDB scores = 1.71, 95% CI: 1.28–2.29, *p* < 0.001), after adjustment for the other variables included in the model. Feed-deficit severity was associated with a higher expected TDB score (ratio of expected scores = 1.15, 95% CI: 1.03–1.28, *p* = 0.015) after adjustment for the other variables in the model. Water-deficit severity was also associated with a higher expected TDB score (ratio of expected scores = 1.10, 95% CI: 1.00–1.21, *p* = 0.048). Standardized herd size was not significantly associated with overall TDB in the adjusted main-effects model (ratio of expected scores = 1.03, *p* = 0.450).

Diagnostic testing confirmed the stability of the primary model despite the strong bivariate correlation between perceived feed and water constraints (*ρ* = 0.93). Variance Inflation Factors (VIFs) for all predictors in the full model remained well below the conventional threshold of 5 (maximum VIF = 2.86 for housing system; feed deficit VIF = 2.36; water deficit VIF = 1.50). Furthermore, sensitivity analyses evaluating feed deficit and water deficit in separate specifications demonstrated remarkable coefficient stability. The expected TDB score ratio for feed deficit in isolation was 1.15 (95% CI: 1.03–1.28), identical to its estimate in the full model (1.15, 95% CI: 1.03–1.28). Similarly, the ratio for water deficit in isolation was 1.10 (95% CI: 1.01–1.21), matching the full model (1.10, 95% CI: 1.00–1.21). Because the conditional estimates did not meaningfully fluctuate across specifications, both variables were retained in the final primary model.

**Figure 6:**
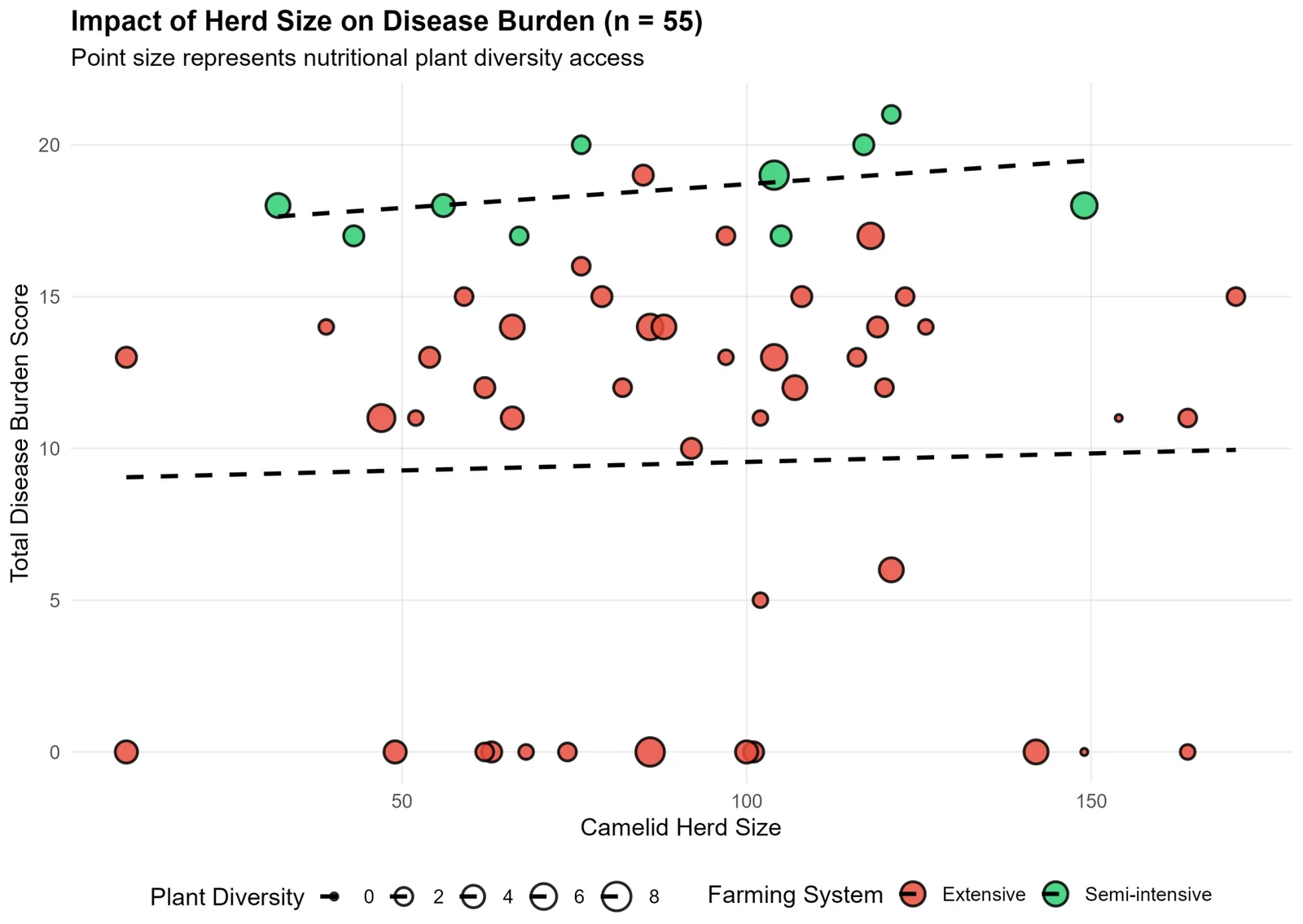
Farm-level Total Disease Burden score in relation to herd size and management system. Each point represents a farm. The horizontal axis shows total herd size (number of camels), and the vertical axis shows the TDB score (0–24). Point size represents the reported diversity of dietary plant taxa. Point colors distinguish extensive, intensive, and semi-intensive management systems. Dashed lines show the displayed trend lines for the extensive and semi-intensive systems; the single intensive holding is presented as an individual observation.

However, exploratory plots stratified by management system suggested that the descriptive relationship between herd size and TDB may differ across systems (Figure 6). Because the primary regression did not include a management-system-by-herd-size interaction term, these patterns should be interpreted as exploratory rather than as evidence of a statistically established interaction. Within semi-intensive herds, TDB scaled positively with herd size, a pattern consistent with a possible density-related association between herd scale and clinical disease burden in confined settings. Conversely, within extensive herds, disease burden remained stable or trended slightly downward with increasing herd scale, with several massive nomadic herds (>100 head) exhibiting near-zero clinical disease.

### 3.6. Animal-Level Morphometrics and Dystocia Associations

**Figure 7:**
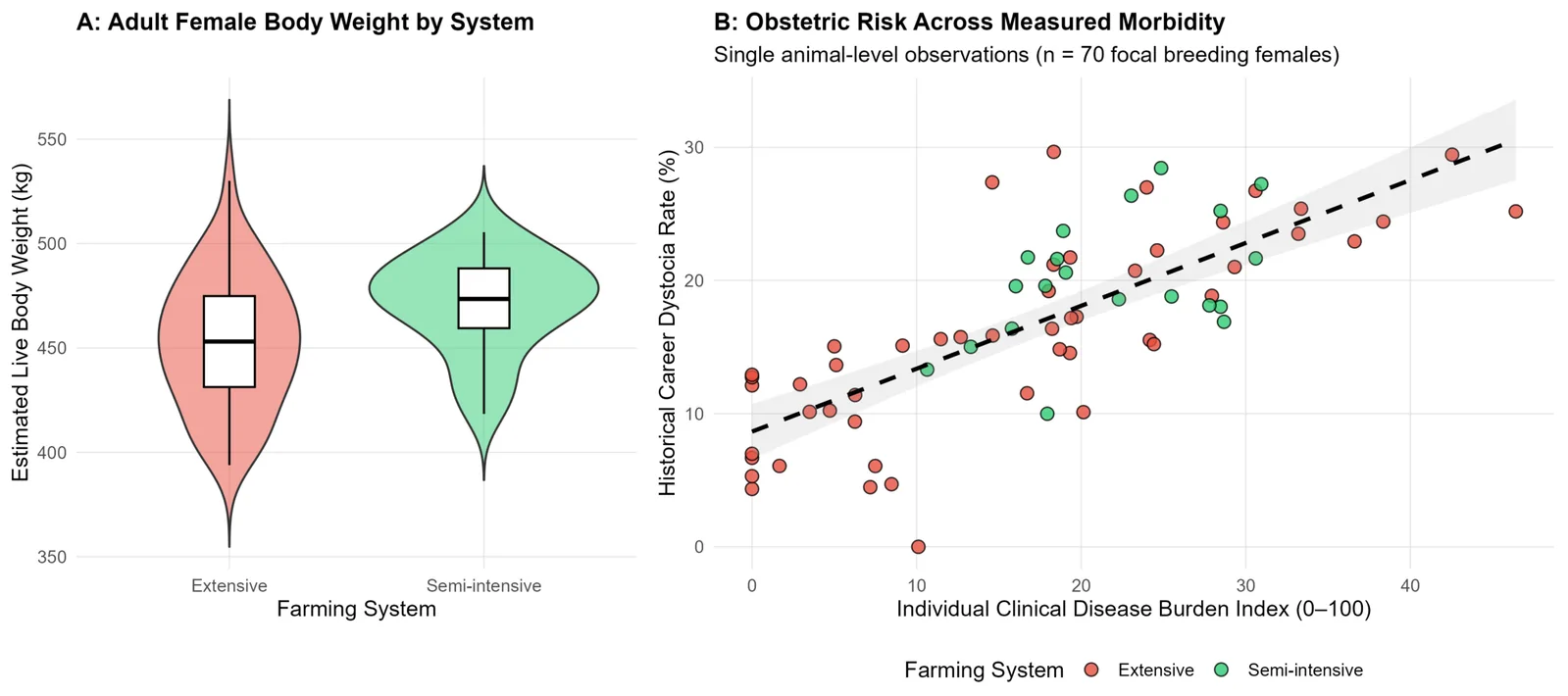
Adult female body weight and obstetric risk. (**A**) Violin and box plots of estimated live body weight (kg) by management system. Box plot hinges represent the 25th and 75th percentiles, the median is indicated by the central horizontal line, and the violin envelope illustrates the probability density of the data. (**B**) Monotonic association (Spearman’s rank correlation) visualizing the positive link between an individual female’s current clinical disease burden (0–100) and her historical rate of calving complications (dystocia rate).

Estimated live body weight was summarized separately for females from extensive, semi-intensive, and intensive holdings (Figure 7A). Among females from extensive holdings, the mean ± SD was 452.3 ± 31.7 kg, compared with 471.3 ± 23.4 kg among females from semi-intensive holdings. In the cluster-aware comparison (linear mixed-effects model with farm as a random effect) of extensive and semi-intensive systems, the estimated difference was 18.2 kg (95% CI: 0.9 to 35.5; *p* = 0.048).

Spearman’s rank correlation was used to assess the association between current clinical DBI and historical individual dystocia rate. Among the 70 focal females, current clinical DBI was strongly and positively associated with the historical proportion of calvings requiring obstetric intervention (Spearman’s ρ = 0.738, *p* < 0.001). To explicitly account for within-farm clustering (non-independence of females sharing the same environment), a farm-level cluster bootstrap confirmed the stability of this relationship (cluster-bootstrapped 95% CI: 0.589–0.826), indicating that the positive correlation remained robust even when adjusting for herd-level sampling (Figure 7B). Females presenting with minimal clinical lesions exhibited low historical dystocia rates, whereas females carrying extensive chronic morbidity exhibited career dystocia rates approaching 30–40%.

### 3.7. Exploratory Predictors of Farm-Level Dystocia

**Figure 8:**
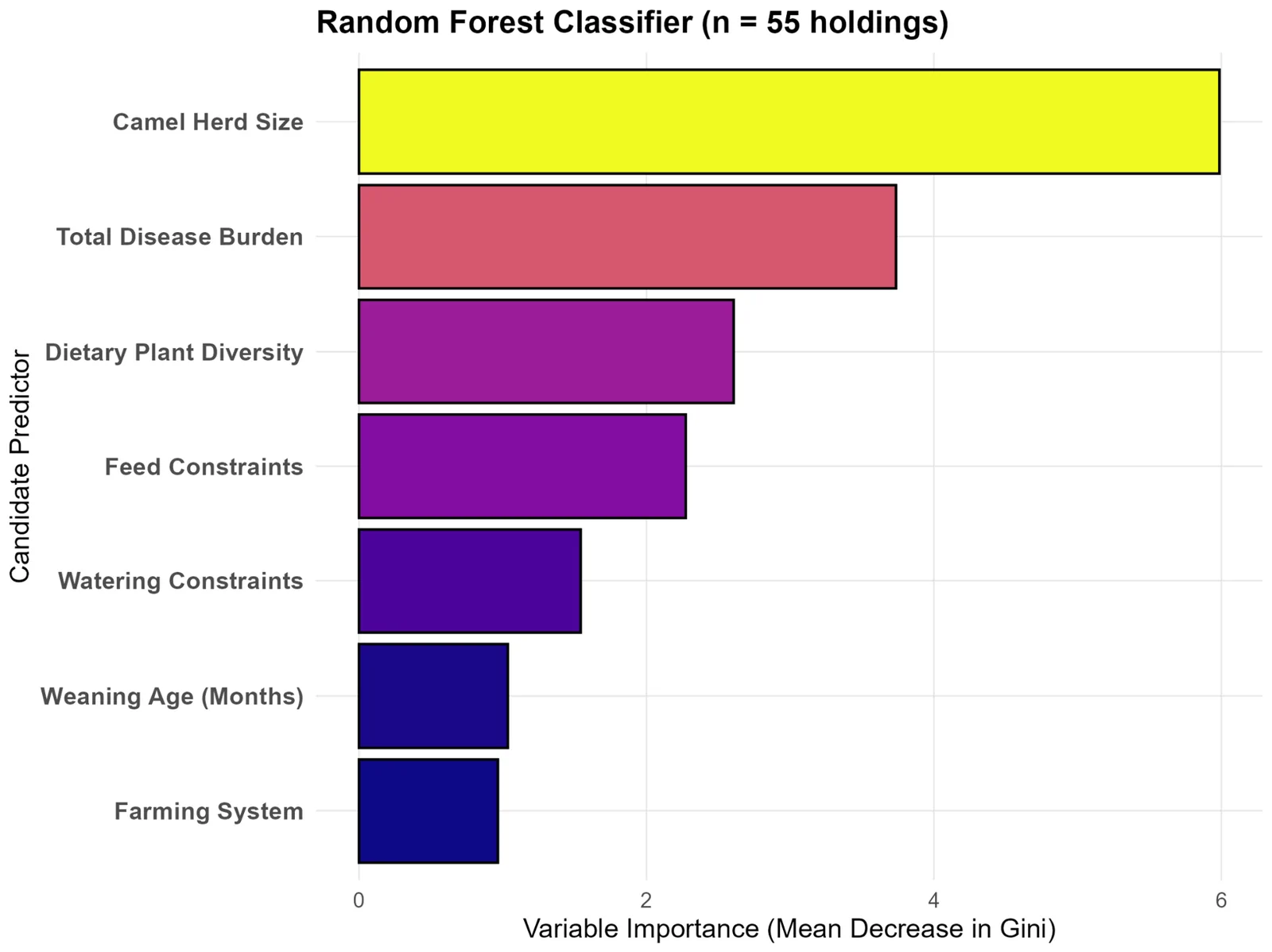
Variable importance in the exploratory Random Forest classification of farm-level dystocia occurrence (n = 55 farms). Bars show the Mean Decrease in Gini for each candidate predictor. The predictors displayed are camel herd size, Total Disease Burden, dietary plant diversity, feed constraints, watering constraints, farming system, and weaning age. The model’s out-of-bag (OOB) classification error was 25.0%.

The exploratory Random Forest classification model for farm-level dystocia occurrence achieved an overall out-of-bag (OOB) error rate of 25.00% (Figure 8). Because dystocia occurred on only 10 of the 55 farms (18.1%), a naive majority-class classifier predicting “no dystocia” for all farms would yield an error rate of 18.1%. The Random Forest OOB error of 25.00% indicates that the model did not achieve stable predictive superiority over this naive baseline.

Consequently, these results are presented strictly to identify variables receiving the highest model-based importance scores in an exploratory classifier, rather than establishing a robust biological hierarchy. Camel Herd Size emerged as the top-ranked variable in the forest structure, closely followed by Total Disease Burden. Dietary Plant Diversity and Feed Constraints provided moderate split improvement. The Farming System label itself ranked significantly lower, suggesting that the structural scale of the herd and its cumulative physiological morbidity correlate more closely with obstetric outcomes than the categorical housing label alone.

## 4. Discussion

This study provides a multi-tiered evaluation of how the structural transition from nomadic pastoralism to sedentary management is associated with changes in the feeding, housing, health, and reproductive management of dromedaries in the Algerian Sahara [8,11]. By structuring the inferential analysis strictly at the farm level, our findings indicate that: (i) environmental and perceived management constraints operate as a tightly coupled syndrome; (ii) semi-intensive confinement is associated with significantly higher observed disease-burden scores independent of herd size; (iii) higher estimated body weight in confined females did not correspond to lower herd-level clinical disease burden; and (iv) herd scale and cumulative disease burden represent the highest-ranked covariates of farm-level dystocia in exploratory classification.

### 4.1. Housing Systems, Density, and Disease Ecology

Semi-intensive management was associated with a ratio of expected TDB scores of 1.71 relative to extensive management, indicating a higher expected composite disease-burden score among the sampled semi-intensive farms after adjustment for the other modeled variables. This empirical finding is consistent with classical epidemiological models of vector ecology in arid environments [29]. The transmission of *Sarcoptes scabiei* var. *cameli* occurs primarily through direct bodily contact or via contaminated physical structures such as rubbing posts, pen fences, and bedding [16]. Similarly, *Hyalomma* ticks proliferate in dry microclimates and accumulate aggressively in permanent earth enclosures where hosts return daily, creating an enduring infestation cycle [17].

Under extensive nomadism, herds may experience greater spatial dispersion across grazing territories, potentially reducing repeated contact with contaminated environments. In contrast, within semi-intensive holdings, disease burden increased with herd size, whereas extensive herds showed comparatively stable disease burden across herd scales. These observations are consistent with a possible density-related association in confined settings, although the observational design does not permit attribution of a causal effect to spatial dispersion itself [1].

Our morphometric data add a critical nuance to welfare assessments. Focal breeding females in semi-intensive and intensive systems had higher estimated live body weights than those in extensive systems [30]. However, this higher estimated live body weight did not correspond to lower herd-level clinical disease burden and should therefore not be interpreted as evidence of superior health. Live body weight in dromedaries is heavily influenced by the provision of energy-dense rations (e.g., barley grain, dry dates) that rapidly expand adipose reserves in the hump and abdominal cavity. This mass gain does not necessarily supply the complex mineral spectrum required for immune function [20]. More broadly, three-dimensional morphometric analysis of dromedary humeri from El Oued has demonstrated substantial shape variation and size-related allometric relationships, illustrating that measurable camel morphology is multidimensional and should not be equated directly with health status [31].

### 4.2. Nutritional Simplification and Hydric Constraints

Feed-deficit severity and water-deficit severity were both associated with higher expected TDB scores in the adjusted model, with ratios of expected scores of 1.15 and 1.10, respectively. The intense coupling of feed deficits and disease reflects the absolute dependence of camelid immune competence on targeted nutrient supply [18]. Deficiencies in essential trace elements—particularly copper, zinc, and selenium—impair phagocytic activity, mucosal integrity, and humoral antibody responses, predisposing animals to severe mange manifestations, secondary bacterial infections, and wasting conditions [19,20]. The relevance of systemic health status is further supported by anatomical and immunohistochemical work in Algerian dromedaries documenting organized splenic lymphoid compartments and substantial populations of immune cells, providing a biological basis for considering cumulative clinical morbidity as more than a collection of unrelated surface syndromes [32,33,34].

The botanical inventory demonstrates the reliance of extensive herds on halophytic Chenopodiaceae (*Anabasis articulata*, *Traganum nudatum*). These halophytes provide massive ash fractions (20–30% DM), sodium, potassium, and chloride, which support ruminal osmotic regulation, fluid balance, and systemic buffering in hyper-arid environments [5,6]. The higher absolute count of feed species in semi-intensive systems is a structural artifact of their management: these farmers actively combine multiple purchased agricultural ingredients (e.g., alfalfa, barley, date by-products) with opportunistic grazing on heterogeneous peri-urban vegetation. In contrast, extensive herds rely exclusively on a narrower, albeit nutritionally specialized, spectrum of native desert halophytes. Crucially, this higher ingredient count in confined herds does not equate to superior nutritional adequacy, as species counts alone do not quantify dietary biomass, intake, seasonal complementarity, or nutrient balance [28].

Furthermore, the positive association for water deficits indicates that hydric restriction acts as a concurrent physiological stressor, compounding herd susceptibility to disease when combined with nutritional scarcity. In the Souf, water scarcity is a feature of the most mobile herds exploiting remote rangelands far from wells, whereas sedentary herds have permanent access to boreholes. Therefore, this coefficient likely reflects structural confounding by pastoral mobility rather than a genuine protective physiological effect of dehydration [3,4].

### 4.3. Obstetric Complications and Dystocia

Dystocia is a catastrophic event in camelid reproduction [23]. In the exploratory Random Forest model, Camel Herd Size and Total Disease Burden emerged as the variables receiving the highest model-based importance scores for farm-level dystocia occurrence.

The prominence of Herd Size suggests a critical limit in supervisory capacity: as herds scale up in transitioning systems, individual veterinary attention drops. In larger herds, unattended calvings may delay critical early obstetric intervention. Without immediate assistance, minor fetal postural abnormalities (such as lateral head deviation or carpal flexion) rapidly progress into severe obstetric impactions, uterine torsion, or uterine rupture requiring surgical delivery [25,26].

The observed associations do not establish the physiological or management mechanisms underlying dystocia. Possible mechanisms include maternal metabolic or mineral imbalances, chronic disease-related debilitation, environmental stress, and delays in detecting or assisting difficult parturition. However, these pathways were not directly evaluated in the present study, because measurements of energy and mineral status, uterine contractility, fetal viability, thermal stress at the individual-animal level, and veterinary response time were not available. Consequently, the following pathways should be interpreted strictly as hypotheses for future targeted research rather than confirmed explanations for the current dataset:

- Nutritional Exhaustion and Uterine Inertia: Chronic ectoparasitism, persistent enteritis, and trace-element deficiencies could contribute to maternal debilitation and deplete metabolic reserves [24]. Energy and calcium–phosphorus imbalances are known to impair myometrial contractions during second-stage labor, potentially leading to primary or secondary uterine inertia [26], although this specific metabolic and contractility pathway was not tested in our sampled animals.
- Subclinical Infections: Pathogens causing systemic debilitation and wasting syndromes might increase the risk of fetal weakness, intrauterine death, or incomplete cervical maturation, which could subsequently predispose dams to fetal malposture and transverse presentations [35]. These pathways remain speculative for the present dataset, as fetal viability and specific etiological pathogens were not directly assessed.
- Chronic Environmental Stress: High disease burdens and physical confinement might increase physiological stress, while concurrent thermal stress could further compromise reproductive performance [22]. Evidence from Algerian livestock demonstrates that elevated temperature–humidity conditions are associated with marked reductions in conception success and increased reproductive effort [36]. In dromedaries, this potential interaction warrants further targeted investigation rather than causal interpretation from the present observational data, as individual thermal stress was not measured directly.

### 4.4. Welfare and Management Implications

These findings identify differences in clinical disease burden and resource constraints that may be relevant to animal health and welfare, although direct validated welfare indicators were not assessed. They highlight the profound risks of directly transferring intensive ruminant husbandry frameworks (e.g., dairy cattle systems) to camel production without accounting for species-specific ethological and physiological mandates [13].

Recent evidence from El Oued further indicates that adaptation of camel husbandry to climate extremes requires coordinated attention to shelter, water availability, forage constraints, veterinary support, and management practices rather than isolated interventions [12]. To support camel welfare and reproductive viability in transitioning Saharan systems, we recommend the following evidence-informed management interventions:

- Density Management, Space, and Parasite Control: Confined holdings should provide adequate space for movement and appropriate housing conditions while implementing targeted hygiene and ectoparasite-control measures adapted to local disease risks [14,16,37].
- Targeted Nutritional Integration: Cultivated rations must be aggressively supplemented with harvested halophytic browse and tailored mineral premixes that specifically match camelid copper, zinc, and selenium metabolism [20].
- Obstetric Protocols and Scaling: Because larger herds are associated with significantly higher dystocia rates, producers scaling their operations must receive intensive training in early obstetric detection, hygiene, and safe traction techniques to prevent mild malpositions from becoming fatal impactions [24,26].
- Multi-Indicator Audits: Future assessments of welfare-related health and management indicators should formally combine morphometric data with rigorous animal-based health metrics, rather than relying on live weight alone.

### 4.5. Limitations

Several methodological constraints frame these findings. First, the 12-month observational and retrospective nature of the dystocia and disease burden data precludes definitive temporal causal sequencing; we present these as powerful, associated overlapping phenomena. Second, the additive TDB index weights all clinical syndromes equally; future iterations could weight diseases by their physiological severity. Third, “wasting compatible with trypanosomosis” was recorded strictly as a field syndromic diagnosis without PCR confirmation, reflecting the realities of Saharan field medicine. Finally, the Random Forest model (OOB = 25.00%) on 55 farms serves purely as an exploratory pattern prioritization tool rather than a robust biological hierarchy or definitive causal proof of intervention effects.

The farm-level constraint scores were semi-quantitative ordinal assessments based on owner reports and field observations. Although the same veterinary investigators conducted the assessments, the absence of independently measured, domain-specific thresholds may have introduced some subjectivity into score assignment. These scores should therefore be interpreted as field-based indicators of perceived and observed constraints rather than as direct quantitative measurements of resource availability or reproductive performance. Furthermore, while the reproductive constraint scale was designed to capture upstream conception and gestation failures, historical dystocia data inherently relied partly on owner recall, which may be subject to information bias.

An additional limitation is the potential for detection and information bias. Clinical disease ascertainment was based on field examination, while selected historical disease and dystocia information relied partly on owner recall and available herd records. Differences in observation intensity, record completeness, or owner recognition of clinical events across management systems may therefore have affected outcome classification. Although the same veterinary investigators conducted the prospective clinical assessments throughout the study period, differential detection across production systems cannot be excluded.

Additional limitations concern the sampling structure, potential selection bias, and the interpretation of outcomes. Farms were selected purposively to represent the observed range of management systems rather than through probability sampling of all camel farms in the region; consequently, the findings may not be generalizable to the wider camel population. Although multiple focal females were sampled from some holdings, the resulting within-farm clustering was explicitly addressed using farm-level bootstrapping and cluster-aware mixed models; however, residual unmeasured confounding shared within environments cannot be entirely excluded. As noted above, farm-level dystocia occurrence did not account for the absolute number of calvings and therefore should not be interpreted as birth-level dystocia prevalence. In addition, the retrospective reproductive records and owner-reported information may have been affected by incomplete recall or documentation. Finally, the timing and nature of the ethics committee’s review are reported transparently in the ethics statement and should be interpreted in accordance with official institutional documentation.

## 5. Conclusions

In the sampled camel farms in the El Oued region of the Algerian Sahara, the transition from mobile pastoralism to semi-intensive management was associated with an unfavorable shift in welfare-relevant health and management indicators. Semi-intensive confinement, compounded by concurrent feed and water deficits, was associated with higher expected composite disease-burden scores (TDB) in the adjusted farm-level analysis, reflecting elevated clinical syndromes such as mange, tick infestations, and wasting. Although confined females exhibited higher estimated live body weights, this mass gain did not correspond to lower herd-level clinical disease burden. Furthermore, farm-level dystocia occurrence was positively associated with both large herd scales and high cumulative disease burdens, pointing to a convergence of possible limitations in supervision—which were not measured directly—and possible maternal exhaustion.

Importantly, these observational findings do not establish that confinement caused the differences or identify the independent biological contributions of correlated resource constraints. The exploratory dystocia classifier did not outperform the majority-class baseline, and farm-level dystocia occurrence could not be interpreted as dystocia risk per birth without complete calving denominators. The findings support further prospective evaluation of management strategies that balance grazing access and confinement with adequate space, nutritional resources, parasite control, and veterinary monitoring, while scaling obstetric supervision appropriately alongside herd expansion. Larger studies incorporating representative farm sampling, standardized disease scoring, complete reproductive records, clustering-aware analyses, and direct welfare indicators are needed to assess these relationships and inform evidence-based camel management.

## Figures and Tables

**Table 1:** Baseline structural and demographic classification of enrolled camel farms (n = 55).

Management System	n Farms	Description & Forage Base	Range of Herd Size	Farms with ≥1 Recorded Dystocia Event, n/N (%)
Extensive	45	Predominantly natural desert rangeland forage, including halophytic and psammophytic taxa.	15–260 head	6/45 (13.3%)
Semi-Intensive	10	Overnight/seasonal confinement; mixed native/cultivated feed.	20–250 head	3/10 (30.0%)

**Table 2:** Field criteria for assigning the 0–3 ordinal constraint scores during farm-level structured interviews.

Score	Feed Availability	Water Availability	Herd Health (Owner-Perceived)	Reproductive Management (Excluding Dystocia)
**0**	**Adequate access:** Abundant natural halophytic pasture or fully sufficient nutritional supplementation year-round; no reported feed shortages.	**Adequate access:** Ad libitum access or daily watering from reliable, clean sources.	**No identified constraint:** Owner reports a robust herd with no recent morbidity or need for veterinary interventions.	**(No identified constraint):** Normal estrus cyclicity, high conception rates, and standard intercalving intervals, with no reported pre-parturient complications.
**1**	**Mild/occasional constraint:** Brief, seasonal periods of forage scarcity during late summer, managed adequately with minor supplementation.	**Mild/occasional constraint:** Watering every 2–4 days (physiologically tolerated) or minor seasonal travel required to reach local wells.	**Mild/occasional constraint:** Sporadic, self-limiting seasonal issues (e.g., mild tick burden) not impacting overall herd productivity.	**Mild/occasional constraint:** Occasional cases of repeat breeding, missed conceptions, or slight extensions of the intercalving interval. (Events are strictly pre-parturient and exclude any term deliveries requiring physical traction or obstetric surgery).
**2**	**Moderate/recurrent constraint:** Prolonged seasonal reliance on overgrazed pasture or low-quality roughage without adequate energy/protein supplementation; observable herd-level body condition decline.	**Moderate/recurrent constraint:** Delayed watering intervals (every 5–7 days) causing visible herd stress, or reliance on highly saline/stagnant surface water.	**Moderate/recurrent constraint:** Recurrent or endemic infectious/parasitic diseases (e.g., symptomatic mange, respiratory signs) requiring frequent traditional or veterinary treatments.	**Moderate/recurrent constraint:** Recurrent pre-parturient reproductive failures, notably prolonged intercalving intervals, or frequent early embryonic loss/abortions occurring prior to full term.
**3**	**Severe/persistent constraint:** Profound lack of accessible pasture and complete absence of nutritional supplementation; severe chronic undernutrition reported.	**Severe/persistent constraint:** Critical water shortages (watering intervals > 7 days) leading to clinical dehydration, or complete failure of water infrastructure forcing emergency relocation.	**Severe/persistent constraint:** Generalized, uncontrolled disease outbreaks, severe cachexia, or significant herd mortality threatening farm viability.	**Severe/persistent constraint:** Pervasive herd infertility, widespread anovulation, or abortion storms fundamentally threatening herd replacement dynamics, entirely independent of term delivery complications.

**Table 3:** Most frequently cited native pastoral taxa utilized by extensive camel herds in El Oued (n = 45).

Vernacular Name	Scientific Taxon	Botanical Family	Citation Prevalence (%)
**Baguel**	*Anabasis articulata* (Forssk.) Moq.	Chenopodiaceae	73.3%
**Demranne**	*Traganum nudatum* Delile	Chenopodiaceae	55.6%
**Helfa**	*Stipa tenacissima* L.	Poaceae	53.3%
**Zita**	*Limoniastrum guyonianum* Boiss.	Plumbaginaceae	44.4%
**Sebat**	*Stipagrostis* spp./*Asphodelus refractus*	Poaceae/Asphodelaceae	40.0%
**Retam**	*Retama raetam* (Forssk.) Webb	Fabaceae	15.6%
**Arta**	*Calligonum comosum* L’Hér.	Polygonaceae	13.3%

**Table 4:** Negative-binomial regression of herd Total Disease Burden (n = 55 farms).

Predictor	Exponentiated Coefficient (Ratio of Expected TDB Scores)	95% CI	*p*-Value
**Semi-intensive vs. extensive**	1.71	1.28–2.29	<0.001
**Feed deficit severity**	1.15	1.03–1.28	0.015
**Water deficit severity**	1.10	1.00–1.21	0.048
**Herd size, per SD**	1.03	0.95–1.12	0.450

Note: TDB is an additive composite score ranging from 0 to 24. Exponentiated coefficients represent ratios of expected TDB scores and are not incidence rate ratios because the model does not include a population-at-risk offset.

## Data Availability

The datasets generated and/or analyzed during the current study are available from the corresponding authors on reasonable request.

## Supplementary Materials

The following supporting information can be downloaded at https://www.mdpi.com/article/10.3390/ani16203303/s1. Table S1: Field criteria for assigning the 0–3 ordinal severity and prevalence scores for the eight clinical syndromes comprising the Total Disease Burden (TDB); Table S2: Baseline reproductive history and dystocia denominators for the 80 focal breeding females included in the individual-level analyses.

## Abbreviations

The following abbreviations are used in this manuscript:

CI	confidence interval
OOB	out-of-bag
DM	dry matter
n/N	sample size vs. population size
