# Supplementary material for: Management System, Resource Constraints, and Composite Disease Burden in Dromedary Camels in El Oued, Algeria: An Observational Study with Exploratory Dystocia Analyses
Source: Animals (Basel). 2026 Oct 20;16(20):3303. doi: 10.3390/ani16203303 (PMC13700740; doi:10.3390/ani16203303)
Supplement: Supplementary file 1 [file animals-16-03303-s001.zip › animals-4606433-supplementary.pdf]

Supplementary Table S1. Field criteria for assigning the 0–3 ordinal severity and prevalence scores for the eight clinical syndromes comprising the Total Disease Burden (TDB).

Clinical Syndrome	0 (Absent)	1 (Sporadic / Mild)	2 (Moderate / Endemic)	3 (Severe / Generalized Outbreak)
**Sarcoptic Mange**	No clinical signs observed in the herd.	Isolated cases with mild localized pruritus/alopecia (e.g., head or neck).	Multiple animals affected with active spreading lesions and hyperkeratosis on flanks or limbs.	Widespread herd infestation with severe diffuse crusting, bleeding, and associated cachexia.
**Tick Infestation**	No macroscopic ticks observed.	Sporadic presence of few ticks (<10 per animal) localized to the perianal or axillary regions.	Endemic moderate burden (10–50 ticks) across multiple body sites on multiple animals.	Massive generalized clusters (>50 ticks per animal) causing visible anemia or tick-worry behavior across the herd.
**Diarrheal Enteritis**	Normal fecal consistency herd-wide.	Transient or mild loose feces in isolated individuals; no systemic signs.	Recurrent watery diarrhea affecting multiple animals; noticeable perineal soiling.	Profuse, widespread watery/mucoid diarrhea with signs of clinical dehydration and risk of mortality.
**Respiratory Disease**	No abnormal respiratory signs.	Occasional serous nasal discharge or sporadic spontaneous coughing.	Endemic mucopurulent discharge, frequent coughing, and tachypnea in several animals.	Severe herd-wide outbreak of dyspnea, severe coughing, pyrexia, and abnormal pulmonary auscultation.
**Skin Necrosis / Abscesses**	No subcutaneous nodules or abscesses.	Rare, small, isolated subcutaneous abscesses on 1–2 animals.	Multiple animals presenting with observable nodular abscessation and peripheral lymphadenitis.	Severe, generalized contagious skin necrosis or massive rupturing abscesses threatening systemic health.
**Musculoskeletal / Lameness**	Normal herd locomotion.	Sporadic mild gait asymmetry not	Endemic joint effusions, moderate	Severe locomotor impairment,

		impeding herd movement.	lameness, or foot-pad ulcerations in multiple individuals.	chronic recumbency, or irreversible lameness preventing grazing.
**Ocular Afflictions**	Clear corneas, no abnormal lacrimation.	Mild, transient serous lacrimation in a few animals.	Noticeable keratoconjunctivitis or purulent lacrimation affecting multiple animals.	Severe outbreak of corneal opacity, ulceration, or blindness affecting herd viability.
**Wasting Syndrome**	Excellent body condition herd-wide.	Sporadic, slight weight loss in isolated older individuals.	Endemic progressive loss of body condition and temporal muscle atrophy in several animals.	Severe, rapid cachexia, extreme emaciation, pale mucous membranes, and high mortality risk.

Supplementary Table S2. Baseline reproductive history and dystocia denominators for the 80 focal breeding females included in the individual-level analyses.

Variable	Summary Statistic
**Age (years)**	8.4 ± 1.9 (Range: 5 - 13)
**Parity**	Median: 3 (IQR: 2 - 4)
**Previous pregnancies**	3.4 ± 1.5
**Previous calvings**	3.4 ± 1.5
**Recorded dystocia events**	Total: 46 events
**Historical dystocia rate (%)**	17.4 ± 6.9
**Missing reproductive history**	0 (0%)
